# Dating the origins of the maize-adapted strain of maize streak virus, MSV-A

**DOI:** 10.1099/vir.0.015537-0

**Published:** 2009-12

**Authors:** Gordon W. Harkins, Darren P. Martin, Siobain Duffy, Aderito L. Monjane, Dionne N. Shepherd, Oliver P. Windram, Betty E. Owor, Lara Donaldson, Tania van Antwerpen, Rizwan A. Sayed, Bradley Flett, Moses Ramusi, Edward P. Rybicki, Michel Peterschmitt, Arvind Varsani

**Affiliations:** 1 South African National Bioinformatics Institute, University of the Western Cape, Private Bag X17, Bellville 7535, South Africa; 2 Institute of Infectious Disease and Molecular Medicine, University of Cape Town, Observatory, Cape Town, South Africa; 3 Centre for High-Performance Computing, Rosebank, Cape Town, South Africa; 4 Department of Ecology, Evolution and Natural Resources, School of Environmental and Biological Sciences, Rutgers University, New Brunswick, NJ 08901 USA; 5 Department of Molecular and Cell Biology, University of Cape Town, Rondebosch, Cape Town 7701, South Africa; 6 Warwick Systems Biology Centre, University of Warwick, Wellesbourne CV35 9EF, UK; 7 South African Sugarcane Research Institute, Mount Edgecombe, KwaZulu Natal, South Africa; 8 Crop Protection, ARC-Grain Crops Institute, Potchefstroom 2520, South Africa; 9 CIRAD, UMR BGPI, TA A54/K, Campus International de Baillarguet, 34398 Montpellier Cedex 5, France; 10 Electron Microscope Unit, University of Cape Town, Private Bag, Rondebosch 7701, South Africa; 11 School of Biological Sciences, University of Canterbury, Private Bag 4800, Christchurch, New Zealand

## Abstract

Maize streak virus (MSV), which causes maize streak disease (MSD), is one of the most serious biotic threats to African food security. Here, we use whole MSV genomes sampled over 30 years to estimate the dates of key evolutionary events in the 500 year association of MSV and maize. The substitution rates implied by our analyses agree closely with those estimated previously in controlled MSV evolution experiments, and we use them to infer the date when the maize-adapted strain, MSV-A, was generated by recombination between two grass-adapted MSV strains. Our results indicate that this recombination event occurred in the mid-1800s, ∼20 years before the first credible reports of MSD in South Africa and centuries after the introduction of maize to the continent in the early 1500s. This suggests a causal link between MSV recombination and the emergence of MSV-A as a serious pathogen of maize.

## INTRODUCTION

Maize streak virus (MSV; family *Geminiviridae*, genus *Mastrevirus*) is amongst the most important plant pathogens threatening food security in Africa (Martin & Shepherd, 2009). Whereas MSV is best known for causing occasionally devastating maize streak disease (MSD) epidemics throughout sub-Saharan Africa, it also infects over 80 other species, primarily wild grasses in the family Poaceae (Damsteegt, 1983; ICTVdb Management, 2006; Konate & Traore, 1992).

Of the 11 known MSV strains (Varsani *et al.*, 2008) only one, MSV-A, is usually found infecting maize plants (Briddon *et al.*, 1994; Hughes *et al.*, 1992; Owor *et al.*, 2007a; Varsani *et al.*, 2008; Willment *et al.*, 2001). MSV-A is also the only MSV strain that causes serious disease in maize (Martin *et al.*, 1999, 2001). Other MSV strains (MSV-B to -K) are generally found infecting wild grass species in the genera *Digitaria* (e.g. MSV-B), *Urochloa* (e.g. MSV-F and -G) or *Setaria* (e.g. MSV-C, -H and -K; Varsani *et al.*, 2008; Rybicki *et al.*, 1998). ‘Grass-adapted’ strains such as MSV-B, -C, -D and -E cannot symptomatically infect any but the most MSV-susceptible maize genotypes (Martin *et al.*, 2001; Schnippenkoetter *et al.*, 2001; Willment *et al.*, 2002).

MSD was first described in 1901 in southern Africa, where it had affected maize since at least the 1870s (Fuller, 1901), suggesting that maize-adapted MSV-A genotypes may have already evolved by the mid-1800s. Maize was only introduced to West Africa by Portuguese traders in the early 1500s and it reached southern Africa in the mid-1600s with the Dutch East India Company (McCann, 2001). It is possible that a grass-adapted progenitor of MSV-A circulating in African grasses during the 16th or 17th centuries was serendipitously capable of causing severe infections in this exotic new host. A second possibility is that grass-adapted MSV-A ancestors progressively adapted to maize over the previous four centuries. A third possibility is that the capacity to cause severe infections in maize arose instantaneously along with the genesis of MSV-A through the chance genetic recombination of two grass-adapted MSV strains. All MSV-A genomes sampled to date are apparently descended from an inter-strain recombinant that derived most of its movement (*mp*) and coat (*cp*) protein genes from an MSV-B-like parental virus, and the remainder of its genome from a parent resembling an ancestral MSV-F and -G-like virus (Varsani *et al.*, 2008). Recombination is extremely common in geminiviruses (Lefeuvre *et al.*, 2009) and has been directly implicated in the emergence of new crop diseases (Fondong *et al.*, 2000; Pita *et al.*, 2001; Monci *et al.*, 2002; García-Andrés *et al.*, 2007a, b). It is particularly plausible that the recombination event that generated MSV-A triggered the emergence of MSV as a serious agricultural pathogen because the event involved the transfer of a mostly self-contained movement and encapsidation ‘module’ (Martin *et al.*, 2005; van der Walt *et al.*, 2008a) that is known to contain the main pathogenicity determinants of MSV-A (Martin & Rybicki, 2002; van der Walt *et al.*, 2008a).

To rationally assess these alternative MSV-A origin hypotheses, it would be useful to know, firstly, when MSV-A diverged from its grass-adapted relatives and, secondly, when the recombination event that produced the ancestral MSV-A occurred. All that is required to meaningfully estimate these dates is a reliable estimate of the MSV evolution rate.

Analyses of mutation frequencies and highly adaptive reversion mutations over very short time periods (<60 days) have indicated that MSV experiences basal mutation frequencies in excess of 2×10^−3^ mutations per site per year (Shepherd *et al.*, 2005, 2006; van der Walt *et al.*, 2009). Longer term monitoring of small MSV populations in individual hosts over 1–6 years (Isnard *et al.*, 1998; Harkins *et al.*, 2009; van der Walt *et al.*, 2008b) has indicated that the MSV substitution rate (the rate at which mutations accumulate in MSV populations over time) is in the range of 2–7×10^−4^ substitutions per site per year (subs per site per year) i.e. approximately three- to tenfold lower than the basal mutation rate.

However, it is presently not known how MSV substitution rate estimates from small experimental populations compare with those from large continent-wide natural populations. Apparent co-divergence of some mastreviruses with their hosts has led to speculation that these viruses have substitution rates of only 10^−8^ subs per site per year in nature (Wu *et al.*, 2008). However, analyses of temporally structured tomato yellow leaf curl virus and East African cassava mosaic virus datasets sampled from nature have indicated global evolution rates for these geminivirus species of between 10^−3^ and 10^−5^ subs per site per year (Duffy & Holmes, 2008, 2009), rates that are broadly concordant with those determined experimentally for both MSV and some tomato yellow leaf curl disease-causing geminiviruses (Ge *et al.*, 2007; Harkins *et al.*, 2009; Urbino *et al.*, 2008).

Here, we describe the application of a Bayesian coalescent-based approach to study temporally structured MSV-A datasets sampled from nature. The methodology used is similar to that recently applied to other important rapidly evolving plant viruses in the families *Potyviridae* (Gibbs *et al.*, 2008) and *Sobemoviridae* (Fargette *et al.*, 2008). Specifically, we infer both the date when the last common ancestor of all currently sampled MSV-A genomes existed and the earliest probable date when the recombination event that generated MSV-A might have occurred. With these estimates in hand, we assess three potential hypotheses of the origin of MSV-A.

## METHODS

### Assembly of temporally structured MSV-A datasets.

We obtained 41 MSV samples archived in freezers between 1979 and 1999 at the Institut de Biologie Moleculaire et Cellulaire du CNRS, Montpellier, France, and the University of Cape Town, South Africa. We cloned and sequenced full genomes from these and an additional 19 genomes sampled between 2005 and 2008 using previously described methods (Owor *et al.*, 2007b; Shepherd *et al.*, 2008; see Supplementary Table S1, available in JGV Online). We combined these sequences with 64 dated sequences taken from GenBank to produce a temporally structured MSV dataset comprising 124 full genome sequences. All of these sequences had been sampled directly from natural environments and none had been passaged for prolonged periods under glasshouse conditions prior to cloning and sequencing. These genomes were aligned together with representative sequences from each of the 10 other known MSV strains using POA (Grasso & Lee, 2004) with manual editing of alignments using mega4 (Tamura *et al.*, 2007).

### Construction of mostly recombination-free datasets.

As recombination can confound substitution rate estimation (Schierup & Hein, 2000), the alignment was analysed, as described by Heath *et al.* (2006), for the presence of recombination using a battery of recombination analysis methods implemented in the program rdp3. Two mostly recombination-free MSV datasets were assembled: (i) comprised 104 sequences each containing ∼900 *mp* and *cp* gene nucleotides (dataset MPCP) and (ii) comprised 125 MSV-A sequences and six MSV-B sequences each containing ∼450 *cp* gene nucleotides (dataset CP). These datasets were only ‘mostly recombination-free’ because despite removing all detectable evidence of recombination, it is very probable that, due to limitations of the methods applied, other very difficult to detect trace recombination signals remained (van der Walt *et al.*, 2009). For the full genome dataset, we removed all nucleotides from predominantly MSV-A sequences that were apparently derived through recombination from other strains. This involved the removal of two short sequence tracts: one of 85 nt from 48 sequences (corresponding to genome coordinates 1346–1431 in MSV-A [Zm-MatA-1994]) and the other of 112 nt from six sequences (corresponding to genome coordinates 155–267 in MSV-A [Zm-MatA-1994]). Except for six MSV-B sequences left in the CP dataset, non-MSV-A sequences were removed from all the datasets following recombination analysis. MSV-B sequences were retained in the CP dataset so that we could later use it to date the recombination event that yielded MSV-A (the tract of MSV-A sequence retained in this dataset was derived from its MSV-B parent). All analysed alignments are available on request.

### Estimation of evolution rates and dating of ancestral sequences.

For all three datasets (whole genome, MPCP and CP) a co-estimate of the nucleotide substitution model [Hasegawa, Kishino and Yano with a calculated proportion of invariable sites with a gamma distribution (HKY+ I+G) or general time reversible (GTR)+I+G] parameters, phylogeny and time to the most recent common ancestor (tMRCA) was obtained for the various major MSV-A lineages using the Bayesian Markov chain Monte Carlo (MCMC) method implemented in beast v1.4.8 (Drummond & Rambaut, 2007). The HKY+I+G_4_ and GTR+I+G_4_ nucleotide substitution models both yielded similar results and we only report here the analyses using HKY+I+G_4._ For each dataset, six different coalescent models were tested including both parametric (constant population size, exponential growth) and non-parametric [Bayesian skyline plot (BSP)] models with both a strict and a relaxed (uncorrelated LogNormal prior) molecular clock.

For each evolutionary model, between 5 and 15 independent runs, each with between 2.0×10^7^ and 5.0×10^7^ steps in the Markov chain were performed using beast and checked for convergence using Tracer v1.4 (Drummond & Rambaut 2007). The effective sample sizes (ESSs) were always >200, indicating sufficient mixing of the Markov chain and parameter sampling. When similar results were produced from independent runs of the Markov chain, the log files were combined with the program LogCombiner v1.4.7 available in the beast package (Drummond & Rambaut 2007).

Identification of the best-fit clock and demographic models was achieved by calculating the Bayes factor (BF), which is the ratio of the marginal likelihoods of the two models being compared (Kass & Raftery 1995; Suchard *et al.*, 2001). This method permits the comparison of non-nested models (such as the non-parametric BSP versus the parametric constant or exponential growth demographic models) that cannot be validly compared using the mean log posterior probabilities.

## RESULTS AND DISCUSSION

### Determining the MSV-A substitution rate

The mean substitution rates estimated for the full genome and mostly recombination-free MPCP datasets with the various clock (strict or relaxed) and demographic (constant population size, exponential growth or BSP) models tested were broadly similar and ranged from 1.8×10^−4^ subs per site per year (for the full genome dataset analysed with the strict clock+BSP model) to 3.9×10^−4^ subs per site per year (for the full genome dataset analysed with the relaxed clock+exponential growth model). For the mostly recombination-free CP dataset, which contained six MSV-B sequences in addition to MSV-A sequences, estimated mean substitution rates were a little higher, ranging from 4.9×10^−4^ subs per site per year (strict clock+constant population size model) to 7.0×10^−4^ subs per site per year (relaxed clock+BSP model). Also, for each individual dataset, 95 % highest probability density (HPD) intervals were largely overlapping irrespective of the demographic and clock model combinations tested. For all three datasets, the BF tests significantly supported models enforcing relaxed over strict molecular clock models (Table 1; note that 2lnBF scores >3 reflect a significantly better fit of model H_1_ versus H_0_). Consistent with these results, the estimated values of the coefficient of variation parameter from the relaxed clock runs (*n*=9 models) ranged from 0.54 to 0.92, suggesting the presence of significant substitution rate heterogeneity among lineages (Drummond *et al.*, 2006). The BF tests also generally supported exponential growth and/or constant population size models over BSP models. For the whole genome and CP datasets, the exponential growth and constant population size relaxed clock models fitted the data equally well, while for the MPCP dataset, both the exponential growth and BSP demographic models fitted the data equally well. We noticed, however, that in the maximum clade credibility trees that we constructed for the CP dataset using the relaxed clock+exponential growth/BSP models, the six MSV-B isolates included in the CP dataset were not basal to the MSV-A sequences. Despite the CP exponential growth and constant population size models being approximately equally supported by BF tests, failure to recover a credible tree under the exponential growth model disqualified this model from further consideration in analyses of the CP dataset.

**Fig. 1. f1:**
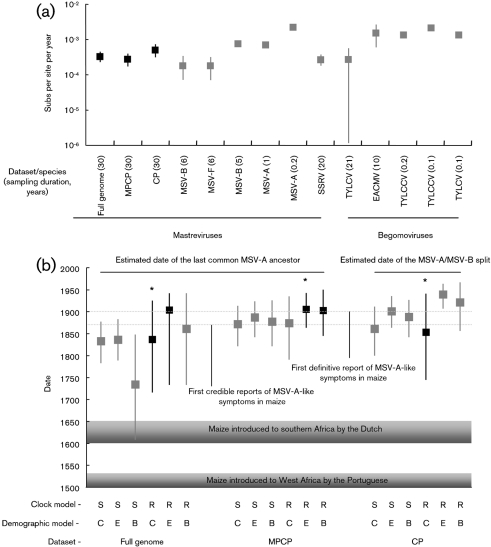
Estimated substitution rates used to date key events in the evolution of MSV-A. (a) Mean substitution rates estimated with temporally structured MSV-A datasets sampled from nature using the best-fit demographic and clock models (highlighted in black) (constant population size+relaxed LogNormal molecular clock for the full genome and CP datasets and exponential population growth+relaxed LogNormal molecular clock for the MPCP dataset) compared with those estimated either experimentally for MSV (van der Walt *et al.*, 2008b; Harkins *et al.*, 2009), sugarcane streak Réunion virus (SSRV) (Harkins *et al.*, 2009), tomato yellow leaf curl China virus (TYLCCV) (Ge *et al.*, 2007) and tomato yellow leaf curl virus (TYLCV) (Urbino *et al.*, 2008) or from temporally structured genome sequence datasets sampled from nature [East African cassava mosaic virus (EACMV) and TYLCV; Duffy & Holmes, 2008, 2009]. Squares indicate most probable substitution rates, vertical bars indicate the 95 % highest probability densities of the substitution rate estimates. (b) Most probable estimates (squares) of dates when the last common MSV-A ancestor existed (estimated with the full genome and MPCP datasets) and when the MSV-A/MSV-B lineages split (indicative of the earliest date when MSV-A could have arisen as estimated with the CP dataset). Vertical lines indicate 95 % HPD intervals, the date estimates determined with the best-fit models (see Table 1) are highlighted in black. The three estimates with the best-fit models that we focus on the most in the text are indicated by asterisks (*). For clock models: S, strict clock; R, relaxed clock. For demographic models: C, constant population size; E, exponential population growth; B, BSP (allowing for varying phases of population growth/decline). Whereas some recombination is detectable in the full genome dataset none was detectable in either the MPCP or CP datasets. The dates of key historical events are indicated for comparative purposes. Dotted lines indicate that a portion of some of the branches and the timeline have been removed for display purposes.

The genome-wide substitution rate that we estimated with the simplest (i.e. least parameter-rich) best fit demographic (constant population size) and clock (relaxed LogNormal) models was 3.5×10^−4^ (95 % HPD interval=2.4×10^−4^–4.6×10^−4^). This rate is very similar to those determined using the same methodology in evolution experiments involving both sugarcane streak Réunion virus (another mastrevirus closely related to MSV) and MSV (Fig. 1a[Fig f1]; Harkins *et al.*, 2009). Importantly, it is also very similar, albeit with much tighter credibility intervals, to genome-wide substitution rate estimates made using tomato-infecting begomoviruses (Duffy & Holmes, 2008). High nucleotide substitutions rates are therefore possibly conserved across the geminiviridae which, despite being DNA viruses, apparently have CP nt substitution rates (5.3×10^−4^) within the range of those observed in the CP genes of rapidly evolving RNA viruses such as sobemoviruses (5.1×10^−4^–12.3×10^−4^ subs per site per year; Fargette *et al.*, 2008) and potyviruses (1.2×10^−4^ subs per site per year; Gibbs *et al.*, 2008).

### Dating the last common MSV-A ancestor

Applying the best-fit clock and demographic models determined for the full genome and MPCP datasets, the most probable dates when the last common ancestor of the MSV-A viruses existed ranged between 1906 (MPCP dataset with exponential population growth+relaxed clock model) and 1839 (full genome dataset constant population size+relaxed clock model; Fig. 1b[Fig f1]). The 95 % HPD intervals for these dates are, however, quite broad, ranging from 1954 (upper limit for the MPCP dataset, BSP+relaxed clock model) to 1716 (lower limit for full genome dataset, constant population size+relaxed clock model; Fig. 1b[Fig f1]).

Despite these broad confidence intervals, this analysis implied that the last common ancestor of all currently sampled MSV-A viruses only arose at least 200 years after maize was first introduced into West Africa by the Portuguese and at least 100 years after it was introduced to southern Africa by the Dutch (McCann, 2001). As there are narrower credibility intervals attained with the recombination-free MPCP dataset (Fig. 1), it is very probable that the last common ancestor of all the MSV-A viruses occurred more recently than 1791 [the lowest 95 % HPD limit for any of the clock and demographic model combinations used to analyse this dataset (constant population size+relaxed clock model)]. Considering the whole genome and MPCP analyses together, this date was most probably between 1839 (full genome+constant population size+relaxed clock) and 1905 (MPCP+exponential growth+relaxed clock); both of these are within 62 years of when MSV was first unambiguously described by Fuller (1901).

### Determining the earliest date when MSV-A could have emerged

While the most recent common ancestor of all currently sampled MSV-A lineages (i.e. the root of the MSV-A phylogenetic tree) probably existed at some time between the mid-1800s and early 1900s, these estimated dates do not necessarily indicate when MSV-A emerged as a serious maize pathogen. The date of this emergence is expected to be older, and to map somewhere on the branch of the phylogenetic tree that separates the MSV-A clade from the other MSV strains. In fact, Fuller (1901) cited reports indicating that something resembling MSD was endemic in South Africa as early as the 1870s, indicating that the origin of MSV-A probably precedes this date.

The earliest possible date for the emergence of MSV-A was when it diverged from its nearest grass-adapted relatives. Estimating this date is paradoxically helped by the fact that all currently sampled MSV-As are apparently recombinants of MSV-B- and MSV-F-like viruses (Varsani *et al.*, 2008). The coat protein gene (*cp*) of MSV-A very closely resembles that of MSV-B, whereas the rest of its genome is derived from a currently unsampled strain most closely resembling MSV-G- or MSV-F-like viruses. The problem of inferring the earliest possible date of emergence of MSV-A is therefore equivalent to inferring the earliest date when the recombination event that yielded MSV-A could have occurred. This is, in turn, equivalent to using the *cp* region of MSV-A and MSV-B viruses to infer the time when the last common ancestor of these strains existed.

By applying an approach to the CP dataset that is similar to that recently used to date the origin of a human immunodeficiency virus circulating recombinant form (Tee *et al.*, 2009), we determined that the last common MSV-A/MSV-B ancestor in the *cp* region most probably existed in 1853 (95 % HPD=17451941; relaxed clock+constant population size model). This indicates that MSV-A and MSV-B share a common ancestor that existed at approximately the same time as the last common ancestor of the MSV-A viruses. In fact, the last common MSV-A/MSV-B ancestor probably did not exist more that 50 years prior to the last common MSV-A ancestor. Considering the lower bounds of 95 % HPD intervals obtained using the best-fit model (relaxed clock+constant population size), this prototypical recombinant MSV-A virus almost certainly existed at some time after 1744 – at least 200 years after maize was first introduced to West Africa and at most 130 years before MSV-A-like infection symptoms were first noted in 1870 by maize farmers in South Africa. It is very interesting that our analysis indicates that 1853 is the most probable date of the MSV-A/MSV-B split, since this implies that the recombination event that produced the first MSV-A genome most probably occurred within 20 years of the first reports of MSD.

### The spread of MSV-A throughout Africa

Given all the available data, we formulated a most-probable timeline for the emergence and spread of MSV-A. Although the recombination event that yielded MSV-A almost certainly occurred between the 1740s and 1870s, it most probably occurred in the 1850s. In the 1870s, symptoms resembling those produced by MSV-A were reported in South Africa, marking this as the latest date at which MSV-A-like viruses might have emerged. Given the high degree of overlap between the estimated dates of the last common MSV-A ancestor and the last common MSV-A/MSV-B ancestor, it is probable that this earliest MSV-A sequence was either reasonably well adapted to maize (possibly due to the initial recombination event that produced it having been selectively favoured in this host) or that its adaptation to maize probably took less than 130 years (the time between the earliest credible date of the MSV-A/MSV-B split and 1870).

**Fig. 2. f2:**
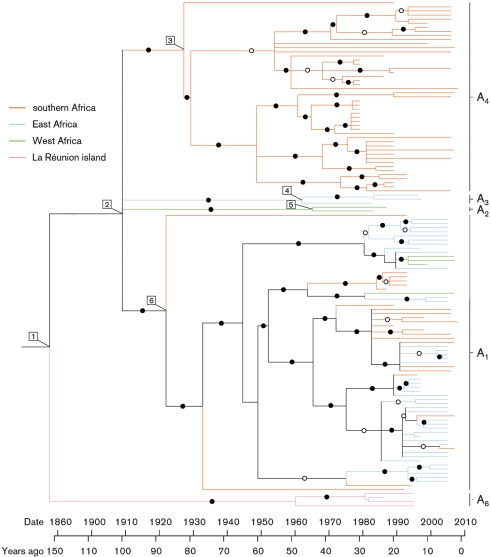
The maximum clade credibility MSV-A phylogenetic tree (for the full genome dataset) recovered under a best-fit demographic (constant population size) and clock (LogNormal relaxed) model. Viruses sampled from southern Africa, East Africa, West Africa and the island of La Réunion are indicated by orange, blue, green and pink branches, respectively. Nodes labelled 1 to 6 indicate the dated most recent common ancestors of major MSV lineages referred to in the text. Branches recovered in >99 % of sampled trees are indicated by filled circles, whereas those recovered in 90–99 % of sampled trees are indicated by empty circles. Branches recovered in less than 80 % of sampled trees were collapsed. An approximate timeline is given beneath the tree and major MSV-A lineages (A_1_, A_2_, A_3_, A_4_ and A_6_, as defined by Owor *et al.*, 2007a) are labelled on the right.

At some time between the 1870s and 1951 (the upper 95 % HPD date considering all best-fit models) and most probably around 1904, an MSV-A variant existed that was to become the last common ancestor of all the MSV-A viruses that have been sampled since 1979 (node 1 in Fig. 2). Whereas the MSV-A_6_ lineage of the descendents of this ancestral virus colonized the Indian Ocean islands off the coast of Africa, another lineage diversified after ∼1904 (95 % HPD=1861–1938; node 2 in Fig. 2) to yield all the MSV-A variants that are currently found throughout mainland Africa.

The most probable date of the MSV-A_6_ split from the mainland MSV-A variants (node 1 in Fig. 2) are between 50 (1870) and 16 (1904) years before the first reliable accounts of MSD on La Réunion island in the 1920s (Kopp, 1930; Kopp & d'Emmerez de Charmoy, 1931, 1932). The most recent common ancestor of all the MSV-A isolates sampled from La Réunion is 1963 (95 % HPD=1945–1978), suggesting that, if these isolates are the descendants of viruses infecting maize on the island in the 1920s, there has possibly been either a reasonably high rate of MSV lineage extinction or a relatively unrepresentative sampling of viruses from this island. It is worthwhile noting here that we recently characterized a slightly more divergent MSV-A_6_ variant from the island of Mauritius that was not included in this study because it had been serially passaged in maize in a glasshouse after sampling (data not shown). This MSV-A_6_ variant nevertheless suggests that a common ancestor of MSV isolates sampled on La Réunion may have been introduced to the island from either Mauritius or some other Indian Ocean island sometime between the 1920s and 1960s.

On mainland Africa, MSV-A spread and differentiated into a southern African lineage (MSV-A_4_) sometime around 1924 (95 % HPD=1893–1949; node 3 in Fig. 2), into an East African lineage (MSV-A_3_) sometime around 1967 (95 % HPD=1939–1980; node 4 in Fig. 2) and a West African lineage (MSV-A_2_) sometime around 1969 (95 % HPD=1940–1981; node 5 in Fig. 2). The last common ancestor of the economically most relevant pan-African lineage, MSV-A_1_, probably existed sometime around 1922 (95 % HPD=1882–1953; node 6 in Fig. 2). Unlike the other MSV-A lineages, the mixing of MSV-A_1_ isolates sampled from East, West and southern Africa within several well-supported clades in the MSV-A phylogenetic tree (Fig. 2), suggests that in the past 80 years, there were multiple waves of continent-wide MSV-A_1_ dispersal.

## CONCLUSIONS

While our results are inconsistent with the first origin hypothesis that MSV-A already existed in Africa at the time when maize was first brought to the continent in the early 1500s, it remains possible that, after MSV-A arose by recombination during the 18th or 19th centuries, it required up to 130 years to adapt to maize before being noticed by farmers in the 1870s (the second origin hypothesis). We have, however, also shown that the recombination event that yielded the MSV-A prototype most probably occurred within 20 years of MSD being first reported. Our data are therefore clearly also consistent with, and strongly supportive of, the third origin hypothesis that the adaptation of MSV-A to maize was very rapid and that the emergence of MSV-A as a serious agricultural pathogen was temporally, and therefore possibly causally, linked with the recombination event that produced this strain.

## Supplementary Material

[Supplementary Table]

## Figures and Tables

**Table 1. t1:** Summary of Bayes factors (BF) tests carried out between different evolutionary models for the full genome, CP and MPCP datasets

**Model comparison**	**Full genome**		**CP**		**MPCP**	
	**2ln BF**	**Evidence against H_0_**	**2ln BF**	**Evidence against H_0_**	**2ln BF**	**Evidence against H_0_**
Const Strict (H_0_) versus relaxed (H_1_) clock	399	Extremely strong	53	Extremely strong	52	Extremely strong
Expo Strict (H_0_) versus relaxed (H_1_) clock	390	Extremely strong	50	Extremely strong	36	Extremely strong
BSP Strict (H_0_) versus relaxed (H_1_) clock	368	Extremely strong	50	Extremely strong	37	Extremely strong
Const (H_0_) versus expo (H_1_) relaxed clock	2	Weak	1	Weak	7	Strong
Const (H_0_) versus BSP (H_1_) relaxed clock	−9	None	−5	None	10	Very strong
BSP (H_0_) versus expo (H_1_) relaxed clock	11	Very strong	6	Strong	2	Weak
