# Supplementary material for: Dating the origins of the maize-adapted strain of maize streak virus, MSV-A
Source: J Gen Virol. 2009 Dec;90(Pt 12):3066–74. doi: 10.1099/vir.0.015537-0 (PMC2885043; doi:10.1099/vir.0.015537-0)
Supplement: [Supplementary Table] [file 0.015537-0_index.html]

 Dating the origins of the maize-adapted strain of maize streak virus, MSV-A -- Harkins et al. 90 (12): 3066 Data Supplement - Supplementary Table -- Journal of General Virology

### Dating the origins of the maize-adapted strain of maize streak virus, MSV-A, by G. W. Harkins, D. P. Martin, S. Duffy, A. L. Monjane, D. N. Shepherd, O. P. Windram, B. E. Owor, L. Donaldson, T. van Antwerpen, R. A. Sayed, B. Flett, M. Ramusi, E. P. Rybicki, M. Peterschmitt and A. Varsani

*Journal of General Virology* vol. **90**, part 12, pp. 3066 - 3074

**Supplementary Table S1.** Sampling locations, dates and full genome sequence accession numbers of maize streak virus isolates examined in this study. [PDF] (294 KB)

  
  
