# Supplementary material for: Dating the origins of the maize-adapted strain of maize streak virus, MSV-A
Source: J Gen Virol. 2009 Dec;90(Pt 12):3066–74. doi: 10.1099/vir.0.015537-0 (PMC2885043; doi:10.1099/vir.0.015537-0)
Supplement: [Supplementary Table] [file 0.015537-0_1.pdf]

Supplementary Table S1. Sampling locations, dates and full genome sequence accession numbers of maize streak virus isolates examined in this study

GenBank accession numbers in bold type are for sequences determined in this study.

Isolate name	GenBank accession no.	Year	Country	Sampling coordinates	
				Latitude	Longitude
MSV-A [BJ-Ben-Mic20-1999]	**FJ882089**	1999	Benin	11.783235	2.727785
MSV-A [KE-Ama-1998]	AF329878	1998	Kenya	–0.066453	36.552286
MSV-A [KE-Gat-1998]	AF329879	1998	Kenya	–0.533094	37.202241
MSV-A [KE-Kag-K14b-1998]	**FJ882090**	1998	Kenya	–0.472354	37.227246
MSV-A [KE-Kan-K4-1998]	**FJ882091**	1998	Kenya	–0.704514	36.980413
MSV-A [KE-Kar-K1-2006]	**FJ882092**	2006	Kenya	–0.511927	37.134254
MSV-A [KE-Ken-1983]	X01089	1983	Kenya	–0.122816	36.829581
MSV-A [KE-MtKA-1997]	AF329885	1997	Kenya	–0.439743	37.127454
MSV-A [KE-Nak-K7-1998]	**FJ882093**	1998	Kenya	–0.262394	36.045339
MSV-A [KE-Oyu-K8-1998]	**FJ882094**	1998	Kenya	–0.492522	34.811004
MSV-A [KE-Sag-1998]	AF329880	1998	Kenya	–0.657416	37.192519
MSV-A [LS-Mal1-Les1-2005]	**FJ882095**	2005	Lesotho	–29.836099	27.631702
MSV-A [LS-Mal2-Les2-2005]	**FJ882096**	2005	Lesotho	–29.839603	27.636714
MSV-A [LS-Mal3-Les3-2005]	**FJ882097**	2005	Lesotho	–29.836535	27.631700
MSV-A [MZ-Bob-g204-2007]	EU628564	2007	Mozambique	–23.713344	32.656117
MSV-A [MZ-Chi4-g210-2006]	**FJ882098**	2006	Mozambique	–24.899634	34.174112
MSV-A [MZ-ChiA-g200-2007]	EU628565	2007	Mozambique	–24.909681	34.191944
MSV-A [MZ-Pem5-Moz41-2007]	**FJ882099**	2007	Mozambique	–13.112033	39.884350
MSV-A [MZ-Pem6-Moz42-2007]	**FJ882100**	2007	Mozambique	–12.966883	39.856867
MSV-A [MZ-Xai1-xaimoz-2007]	**FJ882101**	2007	Mozambique	–24.995472	33.782524
MSV-A [NG-Abe-g239-2007]	EU628566	2007	Nigeria	7.393899	3.875789
MSV-A [NG-Abu1-NG2-2006]	**FJ882102**	2006	Nigeria	9.005609	7.220957
MSV-A [NG-Ile-g82-2007]	EU628567	2007	Nigeria	7.614423	4.179028
MSV-A [NG-Ns-1983]	X01633	1983	Nigeria	7.195348	6.254732
MSV-A [RE-Jos1-Mic18-1995]	**FJ882104**	1995	La Reunion	–21.361408	55.618166
MSV-A [RE-Jos2-Mic19-1995]	**FJ882105**	1995	La Reunion	–21.363003	55.620301
MSV-A [RE-Pie1-Mic1-1986]	**FJ882103**	1986	La Reunion	–21.319311	55.484551
MSV-A [RE-Reu-1995]	X94330	1995	La Reunion	–21.328399	55.512131

Harkins, G. W., Martin, D. P., Duffy, S., Monjane, A. L., Shepherd, D. N., Windram, O. P., Owor, B. E., Donaldson, L., van Antwerpen, T. & other authors (2009). Dating the origins of the maize-adapted strain of maize streak virus, MSV-A. *J Gen Virol* **90**, 3066–3074.

MSV-A [TD-Dja-Mic26-1987]	**FJ882106**	1987	Chad	11.969183	15.262123
MSV-A [UG-Bug248-2005]	EF547118	2005	Uganda	0.253500	32.397667
MSV-A [UG-Bush53-2005]	EF547075	2005	Uganda	–0.614500	30.318333
MSV-A [UG-Hoi154-2005]	EF547099	2005	Uganda	1.444333	31.414333
MSV-A [UG-Hoi159-2005]	EF547102	2005	Uganda	1.637000	31.318000
MSV-A [UG-Iga224-2005]	EF547112	2005	Uganda	0.806833	33.302833
MSV-A [UG-Iga231-2005]	EF547113	2005	Uganda	0.618833	33.448333
MSV-A [UG-Iga235-2005]	EF547114	2005	Uganda	1.147667	34.199667
MSV-A [UG-Jin219-2005]	EF547111	2005	Uganda	0.190833	32.128833
MSV-A [UG-Kab82-2005]	EF547081	2005	Uganda	0.625667	30.224667
MSV-A [UG-KabF48-2005]	EF015782	2005	Uganda	0.625667	30.224667
MSV-A [UG-Kap289-2005]	EF547121	2005	Uganda	1.255500	31.513000
MSV-A [UG-Kap292-2005]	EF547122	2005	Uganda	1.390667	34.488000
MSV-A [UG-KasF42-2005]	EF015780	2005	Uganda	0.013500	29.794000
MSV-A [UG-Kib188-2005]	EF547107	2005	Uganda	0.688000	31.027833
MSV-A [UG-Luw103-2005]	EF547084	2005	Uganda	0.683333	32.523667
MSV-A [UG-Luw107-2005]	EF547085	2005	Uganda	0.895667	32.478000
MSV-A [UG-Luw110-2005]	EF547087	2005	Uganda	0.968333	32.476833
MSV-A [UG-Masin138-2005]	EF547094	2005	Uganda	1.868167	32.052000
MSV-A [UG-Masin139-2005]	EF547095	2005	Uganda	1.970667	32.156833
MSV-A [UG-Masin149-2005]	EF547098	2005	Uganda	1.611667	31.667667
MSV-A [UG-Mask18-2005]	EF547068	2005	Uganda	0.080000	32.129000
MSV-A [UG-Mask21-2005]	EF547069	2005	Uganda	–0.333500	31.706833
MSV-A [UG-Mask23-2005]	EF547070	2005	Uganda	–0.168000	31.682167
MSV-A [UG-Mba41-2005]	EF547074	2005	Uganda	–0.583500	30.523833
MSV-A [UG-MbaF27-2005]	EF015781	2005	Uganda	0.639667	30.595833
MSV-A [UG-Mbal308-2005]	EF547123	2005	Uganda	1.073167	34.117000
MSV-A [UG-Mpi11-2005]	EF547124	2005	Uganda	0.190833	32.128833
MSV-A [UG-Mub94-2005]	EF547066	2005	Uganda	1.868167	32.052000
MSV-A [UG-MubF49-2005]	EF015783	2005	Uganda	0.493500	31.150500
MSV-A [UG-Nak119-2005]	EF547089	2005	Uganda	1.094167	32.539167
MSV-A [UG-Nak120-2005]	EF547090	2005	Uganda	1.094167	32.539167
MSV-A [UG-Nak123-2005]	EF547091	2005	Uganda	1.184167	32.504000
MSV-A [UG-Tor271-2005]	EF547120	2005	Uganda	0.736833	34.090333
MSV-A [UG-Wak4-2005]	EF547064	2005	Uganda	0.282333	32.485300

Harkins, G. W., Martin, D. P., Duffy, S., Monjane, A. L., Shepherd, D. N., Windram, O. P., Owor, B. E., Donaldson, L., van Antwerpen, T. & other authors (2009). Dating the origins of the maize-adapted strain of maize streak virus, MSV-A. *J Gen Virol* **90**, 3066–3074.

MSV-A [ZA-BooB-g145-2006]	EU628570	2006	South Africa	–29.857586	30.950467
MSV-A [ZA-Cpt-M50-1986]	EU628571	1986	South Africa	–33.820419	18.760423
MSV-A [ZA-Emp2-T8-2007]	**FJ882107**	2007	South Africa	–28.769845	31.879080
MSV-A [ZA-Fer-D16-2008]	**FJ882108**	2008	South Africa	–30.081687	30.158735
MSV-A [ZA-Hec1-O4-1989]	**FJ882109**	1989	South Africa	–26.404112	27.596902
MSV-A [ZA-Hec2-O11-1989]	**FJ882110**	1989	South Africa	–26.405819	27.577523
MSV-A [ZA-Hec3-O12-1989]	**FJ882111**	1989	South Africa	–26.400615	27.573666
MSV-A [ZA-Hec4-O13-1989]	**FJ882112**	1989	South Africa	–26.411038	27.593005
MSV-A [ZA-Hec5-O17-1989]	**FJ882113**	1989	South Africa	–26.404078	27.571716
MSV-A [ZA-Hec6-O18-1989]	**FJ882114**	1989	South Africa	–26.404229	27.572284
MSV-A [ZA-Hei-O9-1979]	**FJ882115**	1979	South Africa	–27.003943	27.469688
MSV-A [ZA-Jou1-O25-1989]	**FJ882116**	1989	South Africa	–27.382500	27.114602
MSV-A [ZA-Jou2-O32-1989]	**FJ882117**	1989	South Africa	–27.364935	27.215111
MSV-A [ZA-Koe1-O15-1989]	**FJ882118**	1989	South Africa	–24.912209	27.477556
MSV-A [ZA-Koe2-O21-1989]	**FJ882119**	1989	South Africa	–24.914379	27.477715
MSV-A [ZA-Kom-1989]	AF003952	1989	South Africa	–25.423419	31.917830
MSV-A [ZA-Let1-O8-1989]	**FJ882120**	1989	South Africa	–23.881040	30.441388
MSV-A [ZA-Let2-O10-1989]	**FJ882121**	1989	South Africa	–23.879744	30.419781
MSV-A [ZA-Let3-O14-1989]	**FJ882122**	1989	South Africa	–23.889612	30.429876
MSV-A [ZA-Let4-O16-1989]	**FJ882123**	1989	South Africa	–23.883036	30.419785
MSV-A [ZA-MakD-1989]	AF329884	1989	South Africa	–27.400000	32.200000
MSV-A [ZA-Mal4-T9-2007]	**FJ882124**	2007	South Africa	–25.546119	31.493867
MSV-A [ZA-MitC-g129-2006]	EU628572	2006	South Africa	–29.827092	31.010322
MSV-A [ZA-Nat1-g195-2007]	EU152254	2007	South Africa	–29.425679	30.580311
MSV-A [ZA-Nat2-g194-2007]	EU152255	2007	South Africa	–29.424899	30.580466
MSV-A [ZA-New-D9-2006]	**FJ882126**	2006	South Africa	–29.812606	30.893547
MSV-A [ZA-Omr-g221-2007]	EU628573	2007	South Africa	–29.821500	30.864600
MSV-A [ZA-Por1-D14-2008	**FJ882127**	2008	South Africa	–30.715060	30.398479
MSV-A [ZA-Pot1-Riz48-2007]	**FJ882128**	2007	South Africa	–26.773646	27.127847
MSV-A [ZA-Pot10-O24-1979]	**FJ882129**	1979	South Africa	–26.694789	26.980996
MSV-A [ZA-Pot2-O26-1979]	**FJ882130**	1979	South Africa	–26.672142	26.957923
MSV-A [ZA-Pot3-O27-1979]	**FJ882131**	1979	South Africa	–26.677531	26.988882
MSV-A [ZA-Pot4-O28-1979]	**FJ882132**	1979	South Africa	–26.693178	27.000386
MSV-A [ZA-Pot5-O28k-1979]	**FJ882133**	1979	South Africa	–26.689892	27.029473

Harkins, G. W., Martin, D. P., Duffy, S., Monjane, A. L., Shepherd, D. N., Windram, O. P., Owor, B. E., Donaldson, L., van Antwerpen, T. & other authors (2009). Dating the origins of the maize-adapted strain of maize streak virus, MSV-A. *J Gen Virol* **90**, 3066–3074.

MSV-A [ZA-Pot6-O29-1979]	**FJ882134**	1979	South Africa	–26.726059	27.000132
MSV-A [ZA-Pot7-O31-1979]	**FJ882135**	1979	South Africa	–26.741480	26.974811
MSV-A [ZA-Pot8-O33-1979]	**FJ882136**	1979	South Africa	–26.697977	26.938333
MSV-A [ZA-Pot9-O34-1979]	**FJ882137**	1979	South Africa	–26.708951	27.033206
MSV-A [ZA-Ros-D2-2006]	**FJ882138**	2006	South Africa	–29.881770	30.959587
MSV-A [ZA-RosB-g142-2006]	EU628574	2006	South Africa	–29.895572	30.972438
MSV-A [ZA-RosE-g131-2006]	EU628575	2006	South Africa	–29.895567	30.972435
MSV-A [ZA-SA-1986]	Y00514	1986	South Africa	–26.768834	26.828745
MSV-A [ZA-ThoE-g132-2006]	EU628568	2006	South Africa	–29.902700	31.032889
MSV-A [ZA-TreA-g141-2006]	EU628569	2006	South Africa	–29.960970	30.977001
MSV-A [ZA-VM-1993]	AF239961	1993	South Africa	–28.565084	24.587404
MSV-A [ZA-Wil-O38-1993]	**FJ882139**	1993	South Africa	–33.964583	22.585376
MSV-A [ZW-Har2-Mic22-1987]	**FJ882140**	1987	Zimbabwe	–17.654387	31.068617
MSV-A [ZW-Hel2-Bet36-2006]	**FJ882141**	2006	Zimbabwe	–17.699683	31.124733
MSV-A [ZW-Mas1-Bet43-2006]	**FJ882142**	2006	Zimbabwe	–19.763083	31.200950
MSV-A [ZW-Mas2-Mic4-1993]	**FJ882143**	1993	Zimbabwe	–20.176270	30.641234
MSV-A [ZW-Mas3-Mic5-1993]	**FJ882144**	1993	Zimbabwe	–20.307354	30.823370
MSV-A [ZW-Mas4-Mic6-1993]	**FJ882145**	1993	Zimbabwe	–20.305141	30.764917
MSV-A [ZW-Mas5-Mic7-1993]	**FJ882146**	1993	Zimbabwe	–20.357616	30.620880
MSV-A [ZW-Mas6-Mic8-1993]	**FJ882147**	1993	Zimbabwe	–20.215719	30.697400
MSV-A [ZW-MatA-1994]	AF329881	1994	Zimbabwe	–21.368032	30.891077
MSV-A [ZW-MatB-1996]	AF329882	1996	Zimbabwe	–21.413335	30.786315
MSV-A [ZW-MatC-1989]	AF329883	1989	Zimbabwe	–21.390356	30.832604
MSV-A [ZW-Maz3-g265-2008]	**FJ882148**	2008	Zimbabwe	–17.703186	30.925806
MSV-A [ZW-Maz4-Mic3-1992]	**FJ882149**	1992	Zimbabwe	–17.772084	31.265549
MSV-A [ZW-Nmg-g168-2006]	EU628576	2006	Zimbabwe	–17.762852	31.085185
MSV-A [ZW-Nmg-g186-2006]	EU628576	2006	Zimbabwe	–17.799204	31.091842

Harkins, G. W., Martin, D. P., Duffy, S., Monjane, A. L., Shepherd, D. N., Windram, O. P., Owor, B. E., Donaldson, L., van Antwerpen, T. & other authors (2009). Dating the origins of the maize-adapted strain of maize streak virus, MSV-A. *J Gen Virol* **90**, 3066–3074.
